# scplainer: using linear models to understand mass spectrometry-based single-cell proteomics data

**DOI:** 10.1186/s13059-025-03713-4

**Published:** 2025-08-07

**Authors:** Christophe Vanderaa, Laurent Gatto

**Affiliations:** Computational Biology and Bioinformatics Unit (CBIO), de Duve Institute, UCLouvain, Av. Hippocrate 75, 1200 Brussels, Belgium

**Keywords:** Single-cell, Mass spectrometry, Proteomics, Data analysis, Reproducible research, Linear modeling, Data interpretation, Missing values, Batch correction

## Abstract

Analyzing mass spectrometry (MS)-based single-cell proteomics (SCP) data faces important challenges inherent to MS-based technologies and single-cell experiments. We present *scplainer*, a principled and standardized approach for extracting meaningful insights from SCP data using minimal data processing and linear modeling. scplainer performs variance analysis, differential abundance analysis, and component analysis while streamlining result visualization. scplainer effectively corrects for technical variability, enabling the integration of data sets from different SCP experiments. In conclusion, this work reshapes the analysis of SCP data by moving efforts from dealing with the technical aspects of data analysis to focusing on answering biologically relevant questions.

## Background

Mass spectrometry (MS)-based single-cell proteomics (SCP) has been enabled thanks to impressive technological advancements pioneered by various research groups [1–5]. These breakthroughs have resulted in a broad landscape of SCP methodologies that quantify thousands of proteins at single-cell resolution. Despite these remarkable achievements, the field currently lacks dedicated computational methods to leverage the information contained in the data generated by these cutting-edge technologies. The task of uncovering meaningful biological knowledge from quantitative SCP data is a considerable challenge that requires overcoming several obstacles.

Various protocols exist to perform SCP experiments. For example, some sample preparation protocols perform sample multiplexing by including a labeling step (e.g., with TMT or mTRAQ), while other protocols perform label-free sample preparation and acquire a single cell per run. Additionally, MS instruments may acquired data using data-dependent acquisition (DDA), data-independent acquisition (DIA), or more recently wide-window acquisition (WWA, [6]). The diversity in sample preparation and data acquisition protocols introduces distinct data types with different peculiarities. In previous work [7], we surveyed the current practices of SCP data analysis and found that most data processing workflows are poorly justified, tend to be overly complex, and are often tailored to specific data sets. This hinders the reuse of data analysis pipelines across experiments, leading each lab to analyze their data with ad hoc workflows that lack methodological validation. Consequently, there is a pressing need to develop principled SCP data processing approaches that are applicable off-the-shelf, thoroughly validated, and capable of accommodating the diverse range of SCP data types.

SCP data are characterized by important batch effects [8]. These batch effects arise from inherent fluctuations during cell culture, sample preparation, and data acquisition. While they are inevitable, they can be computationally removed upon data modeling, provided that the sources of batch effects are properly documented during the experiment. SCP data are also characterized by a high proportion of missing values. The prevailing approaches in SCP data analyses include the imputation of missing values with estimates. However, imputation introduces bias, ignores the underlying uncertainty of estimation, and masks inestimable contrasts [9]. Batch effects and missing values are not independent and they should be concurrently addressed. Therefore, the second need is to develop an approach that disentangles the technical variability from the biological variability while explicitly accounting for the presence of missing values.

SCP data, similar to most single-cell omics data, are complex and large. They contain information for thousands of peptides/proteins and hundreds to thousands of single cells, and the numbers are likely to grow in the future. Achieving a deep understanding of the underlying processes present in the data poses a considerable challenge. Data models that yield interpretable and explorable results offer a valuable approach to gaining a comprehensive understanding of the data. Interpretable results mean that meaningful insights can be derived without ambiguity, allowing to answer biological questions while ensuring and validating that undesired technical effects are controlled. Explorable results mean that these insights are easily accessible through structured data tables and flexible visualization. Consequently, the third need is to develop tools that generate interpretable and explorable results.

Different SCP experiments aim to answer different research questions, thereby requiring different downstream analysis methods. For instance, if the objective is to identify clusters of co-regulated proteins, the downstream analysis may consist of a correlation analysis [10]. Conversely, when investigating the differentiation of a cell type into another, trajectory analysis is preferred [11, 12]. Many downstream methods, such as clustering or trajectory inference, are readily available from the scRNA-Seq field [13, 14]. Hence, the fourth need is to develop software that rely on standardized data formats, thereby streamlining the integration with existing methods to perform downstream analyses.

Finally, the fifth need is the implementation of an approach in an open-source and well-documented software package that allows for reproducible results. Open-source software promotes trust by opening implementation details to the user’s scrutiny and hence fosters community contributions and improvements. High-quality documentation is essential for wider adoption of the approach by new users. Reproducibility increases the credibility of the discovery and facilitates the adoption of the approach by other research.

This work introduces a solution to tackle all the needs. We will begin by describing the data processing and modeling approach. We will then explore the modeling results using three complementary approaches. Next to that, we will illustrate how our solution can be used to integrate data sets acquired from different experiments. Finally, we will benchmark our method against popular batch correction approaches on SCP data.

## Results

### A standardized workflow for SCP data analysis

**Fig. 1:**
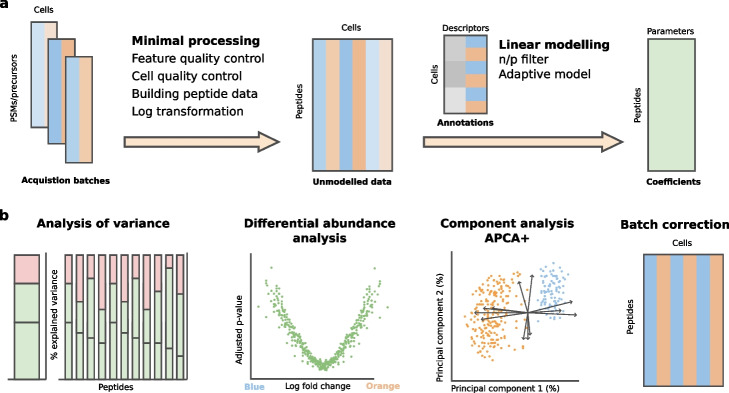
The scplainer workflow. **a** Conceptual overview of the data processing and modeling. The workflow starts with quantified PSM or precursor data obtained from different MS acquisition batches. The data undergo a minimal processing, generating quality-controlled, log-transformed peptide data. Subsequently, a linear model estimates coefficients by fitting user-provided descriptors to the peptide data. The different colors represent different cell types, while the different shades represent batch effects. **b** The coefficients are explored using three methods: analysis of variance, differential abundance analysis, and component analysis. The model output can also generate batch-corrected data for further exploration through downstream analyses

We present scplainer, an SCP-based Linear modeling Approach for Interpretable aNd Explorable Results. At its core, the approach performs statistical modeling using linear regression (Fig. 1a). Linear regression was chosen due to its flexibility, interpretability, and widespread use in omics data analysis [15]. The model takes a matrix of peptide intensities across single cells and a table with cell descriptors. These descriptors convey information about known biological conditions, like cell type or treatment, and technical factors, like the MS acquisition run or the sample preparation batch, that influence the peptide intensities [16]. The descriptors are converted into model parameters and, subsequently, the intensity data are regressed on these parameters to derive estimated coefficients (Fig. 1a, right).

The estimated coefficients hold interesting properties. Firstly, as the coefficients contain the contribution of each parameter, we can assess the proportion of the variation in the data attributed to each factor independently. Secondly, when the model contains both biological and technical descriptors, it disentangles biological effects from unwanted technical effects, leading to effective normalization and batch correction. Finally, the coefficients are highly interpretable. For categorical descriptors like cell type, the coefficients provide the fold changes in peptide intensity between groups of interest. For numerical descriptors like treatment concentration or cell size, the coefficients provide the strength of the relationship between the descriptors and the peptide intensity. A multivariate analysis of the coefficients across peptides using principal component analysis (PCA) facilitates the exploration of correlation patterns, leading to a deeper understanding of the features that drive cellular heterogeneity.

Accurate data modeling depends on quality data. Therefore, a data processing workflow is often carried out before data modeling. While most SCP data processing workflows include batch correction, normalization, protein aggregation, and imputation steps [7], we intentionally omitted these steps from data processing for two reasons. Firstly, the challenges addressed by these steps are often interdependent, meaning that the order of the steps matters and there is no guarantee that any order is suitable. For instance, missing value patterns are influenced by batch effects [8]. Consequently, batch correction after imputation is not equivalent to imputation after batch correction [17] (Fig. S1). Secondly, these steps involve an estimation process with associated uncertainty. However, this uncertainty is lost and hence ignored when the data are transformed sequentially. Therefore, we designed a minimal data processing workflow to minimize the risk of processing artifacts [7] (Fig. 1a, left). It consists of four well-justified steps: feature quality control, sample quality control, precursor-to-peptide aggregation, and log transformation.

To account for the presence of batch effects, we recommend modeling descriptors associated with biological groups concurrently with descriptors related to sources of batch effects. This enables an effective batch correction while preserving biological information. Additionally, it ensures an accurate estimation of the degrees of freedom required for statistical inference.

We also recommend performing cell-wise normalization during modeling to facilitate the exploration of normalization effects. Normalization is modeled by including a cell normalization factor as a technical descriptor. Cell normalization factors can be easily derived from the data, one prevalent approach being the use of the median intensity for each cell [7]. We found that including normalization during modeling does not impact the separation of biological groups nor the mixing of batch effects when compared to normalization during processing (Fig. S3). In addition to cell-wise normalization, several studies include a peptide- or protein-wise normalization step during data processing, presumably to account for the differences in ionization efficiency [7, 11, 18–20]. scplainer estimates a baseline intensity for each peptide, constituting a form of peptide-wise normalization.

In addition, we avoid a protein aggregation step and opt to model intensities at the peptide level instead. Mapping peptides to proteins is not trivial and may potentially obscure biological variation [21, 22]. For added convenience, scplainer provides tools to combine peptide-level results into protein-level results (see the “Methods” section).

Since scplainer relies on linear regression, any missing value will be ignored, alleviating the need for data imputation. However, the patterns of missing values can reduce the number of groups in a descriptor. For instance, some peptides may only be found in a single sample preparation batch or across only a limited number of MS acquisition batches. Hence, the model is adapted to only include the parameters related to these batches, effectively accounting for batch-induced patterns of missing value (see the “Methods,” “Linear modeling” section). Given that different peptides exhibit distinct patterns of missing values and hence a different model to estimate, the number of parameters varies across peptides. We took advantage of this property to develop a new filtering criterion when removing highly missing peptides. Peptides or protein groups are usually filtered based on a user-defined proportion of missing values. Defining a cut-off is subject to arbitrary choices, with no clear guidelines [23]. For instance, the pepDESC approach suggests removing peptides with more than 60% missing values [21]. While this may be a reasonable cut-off for bulk proteomics data [24], this leads to a dramatic data loss in SCP applications (Fig. S2). Instead, scplainer removes features for which there are not enough observations to confidently fit the model. This can be assessed by computing the *n* over *p* ratio (*n*/*p*), that is the number of cells for which there is a measured intensity divided by the number of coefficients to estimate for a peptide. Any peptide that has an *n*/*p* lower than 1 cannot be modeled and should be discarded. We observed that filtering out peptides with the widely used 95% missing value threshold [18, 25] removes more peptides than filtering out peptides with $n/p <= 1$ (Fig. S2).

We recommend using this minimal processing workflow and making use of data modeling to explicitly account for additional processing needs such as addressing batch effects and normalization. However, scplainer is modular and flexible, allowing users to add, remove, or replace steps based on their specific requirements (see the “Methods,” “Code and data sets” section). It is essential to note that scplainer does not enforce a fixed set of descriptors for modeling. For instance, when analyzing the plexDIA data set from Leduc et al. [18], which contains no known sources of biological effects, the model only includes technical variables (Table S1). Similarly, the model used to analyze the data set from Woo et al. [26] contains no descriptor about cell labeling since the data were acquired by a label-free technology (Table S1). Hence, the model specification can be adapted to the available information.

Once the data are modeled, the filtered, normalized and batch-corrected data can be retrieved for further downstream analysis, such as clustering or trajectory inference. Here, we will expand on how to explore SCP data through the output of the linear regression model. The model exploration consists of three complementary approaches (Fig. 1b): analysis of variance, differential abundance analysis, and component analysis.

### Data exploration through analysis of variance

**Fig. 2:**
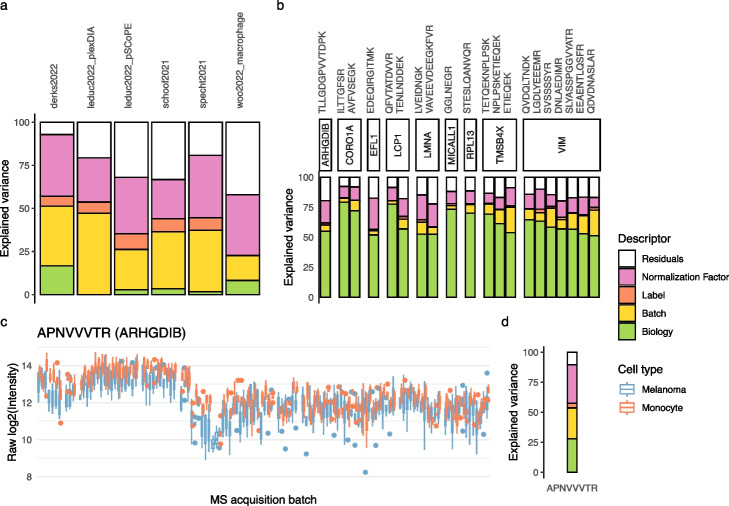
Analysis of variance. **a** The contribution of each model descriptor is shown for different data sets. See Table S1 for more details about the data sets used. No biological descriptor is available for the plexDIA data set published by Leduc et al. [18]. Similarly, no label descriptor is available for the label-free data set published by Woo et al. [26]. **b** The contribution of each model descriptor is shown for the top 20 peptides with the highest biological variance, focusing on the data set from Leduc et al. [18]. Peptides are grouped based on their parent protein. **c** Distribution of the log2 intensities for the APNVVVTR peptide in the data set from Leduc et al. [18]. Single cells are grouped within a boxplot based on the MS acquisition run (sorted by the moment of acquisition) and colored by cell type. **d** Analysis of variance for the peptide presented in **c**

The analysis of variance quantifies the amount of information captured by each cell descriptor in the model. Across all data sets tested, technical descriptors predominantly contribute to the data, with batch effects and normalization contributing equally (Fig. 2a). This observation highlights the importance of thoroughly identifying and documenting potential sources of batch effects [16], and normalization alone is not sufficient to remove technical variations in an experiment. In multiplexed experiments, a small portion of the variance is explained by the labeling effects, further emphasizing the importance of documenting and including the label descriptor in the model. Additionally, the model fails to capture a noticeable proportion of the variance. These model residuals are the consequence of noise in the data, but also the result of unmodeled effects. We will later explore the residuals to uncover unknown sources of biological effects.

Known biological factors capture only a small proportion of the variance (Fig. 2a). A data set with limited biological variation does not necessarily indicate a failed or poor-quality experiment. For the remainder of this analysis, we will take the data set published by Leduc et al. [18] as a use case (Table S1). In this data set, only about 3% of the total variance is explained by the cell type (Fig. 2a). However, small biological effects do not imply the absence of interesting biological information to retrieve.

The first evidence for the presence of biologically-relevant information appears when exploring the variance explained in individual peptides. We find peptides for which most of the variance is explained by the cell type (Fig. 2b). This was already observed in longitudinal multi-omics data using the PALMO framework [27]. The peptides with the highest biological variance are mainly associated with proteins involved in cytoskeleton rearrangements (CORO1A, LMNA, TMSB4X, VIM). While the identification of cytoskeleton proteins might suggest potential confounding effects stemming from differences in cell size, we can exclude this hypothesis based on the observation that normalization effects are correlated with the cell diameter (Fig. S4a). Moreover, despite variations in cell diameters between melanoma cells and monocytes, the intensity within each cell type shows no correlation with cell diameter (Fig. S4b). Together, these observations indicate that the normalization performed by the model effectively corrects for differences in cell size.

The second indication for interesting biological effects in the data is that several peptides bear biological information even though the majority of their variance is explained by technical effects. One of the peptides for which most of the variance is explained by the cell type belongs to the Rho GDP-dissociation inhibitor 2 (encoded by ARHGDIB, Fig. 2b). Consequently, we expect the other peptides that belong to that protein to also bear biological information. Figure 2c shows the intensity distributions for one of these peptides. The peptide is strongly influenced by technical effects and only about 25% of the variance is explained by the cell type (Fig. 2d). However, we still observe a consistent and systematic increase in intensity in melanoma cells. In conclusion, strong batch effects do not necessarily harm the quality of a data set, provided that the experimental design allows for accurate modeling and correction of these batch effects.

### Data exploration through differential abundance analysis

**Fig. 3:**
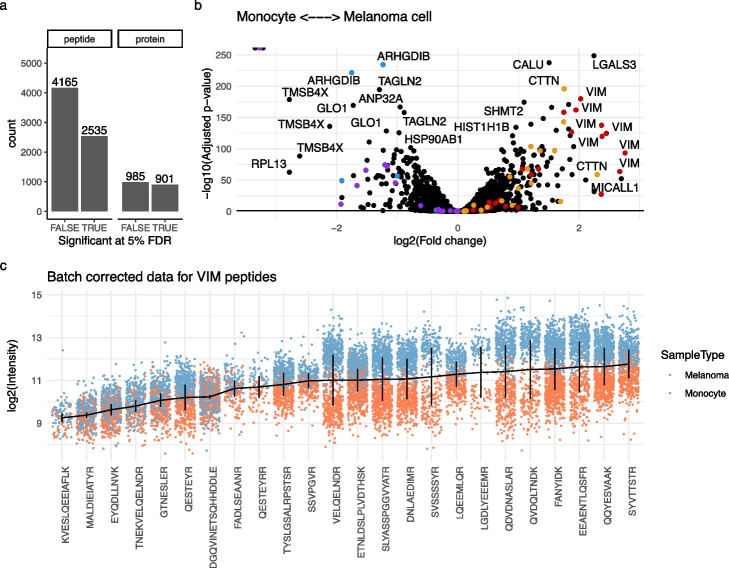
Differential abundance analysis on the data set by Leduc et al. [18]. **a** Summary of the statistical inference at the peptide or protein level. Counts are the number of peptides/proteins that are significant (TRUE) or not (FALSE) at a 5% FDR threshold. **b** Volcano plot that shows the adjusted *p*-values against the log fold change between melanoma cells and monocytes. Each point represents a peptide. A positive log fold change indicates higher abundance in melanoma cells. To illustrate the consistency of the statistical inference, the peptides belonging to four proteins are highlighted: VIM (red), CTTN (orange), ARHGDIB (blue), and LCP1 (purple). **c** The intensity distribution of the 20 peptides mapped to VIM. The data are batch-corrected, that is we retained the effect of cell type, the baseline intensity (intercept) and the residual data. Each point represents a single cell and is colored by cell type. The horizontal lines highlight the modeled baseline (intercept) and the vertical lines connect the average intensity between the cell types

Differential abundance analysis delves deeper into the exploration and understanding of biological effects by estimating the magnitude of change in peptide intensities and its significance between groups of interest, for example, melanoma cells and monocytes. Out of the 6700 modeled peptides, 2535 are significantly differentially abundant (Fig. 3a). When there is no biological difference, i.e., by randomly splitting monocytes into two groups, the *p*-value distribution is uniform, supporting the validity of our statistical inference procedure (Fig. S6). After combining the results at the protein level (see the “Methods,” “Differential abundance analysis” section), 901 out of the 1886 proteins show significant differential abundance. These results further support that the model can confidently retrieve biological information despite the strong batch effects. Notably, proteins associated with cell adhesion and mobility, such as VIM, CTTN, and LGALS3, are more abundant in melanoma cells. Conversely, TMSB4X, known for sequestering actin and hence reducing cellular rigidity, is more abundant in monocytes (Fig. 3b). This sheds light on the morphological differences between the two cell types during cell culture, that is melanoma cells are adherent while monocytes grow in suspension.

The estimated fold changes are consistent for peptides belonging to the same protein. For instance, all peptides that originate from VIM or CTTN are consistently more abundant in melanoma cells while peptides from LCP1 and ARHGDIB are consistently more abundant in monocytes (Fig. 3b), although the magnitudes are influenced by the baseline peptide intensity. Focusing on the vimentin peptides (Fig. 3c), the baseline intensities span up to three orders of magnitude. We notice that differences between cell types tend to be smaller when the baseline is lower. However, we also see variability for log fold changes with similar baselines, as is the case for peptides QESTEYR and DGQVINETSQHHDDLE. This variability could be influenced by technical factors such as peptide co-isolation, false peptide identifications, ambiguity of peptides belonging to multiple proteins, or the presence of protein isoforms such as post-translational modifications or splicing variants [28, 29]. This underscores the importance of modeling the data at the peptide level for a fine-grained understanding of biological processes.

### Data exploration through component analysis

Variance and differential analysis, while valuable, may not fully capture the intricacies of single-cell applications as they do not explore the cellular heterogeneity. Component analysis provides a powerful approach by condensing high-dimensional data into a few informative dimensions for visual exploration. scplainer integrates component analysis with linear regression thanks to the ANOVA principal component analysis extended to linear models (APCA+) framework ([30], see the “Methods” section), where PCA is performed on each modeled effect in the presence of residual data.

**Fig. 4:**
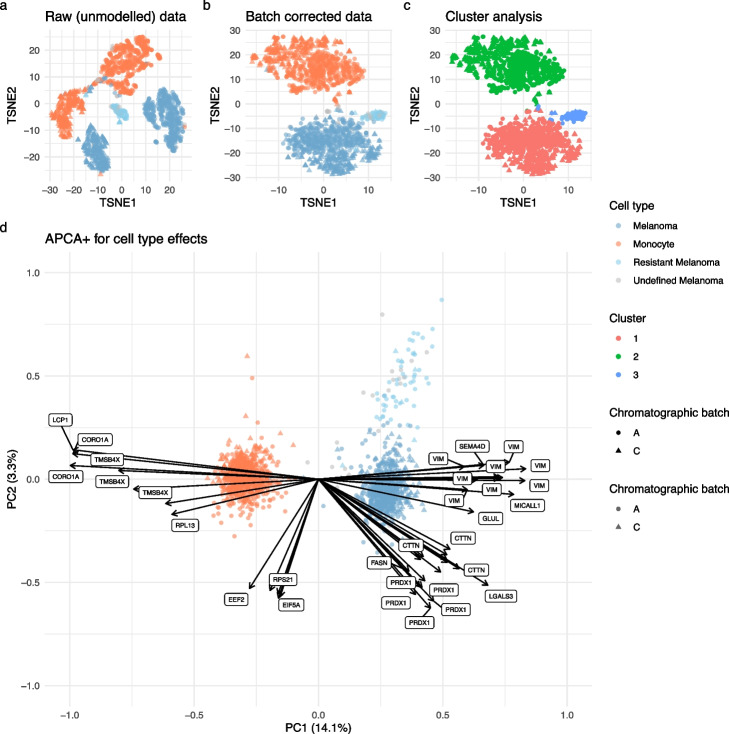
Component analysis on the data set by Leduc et al. [18]. **a** t-SNE of the data before data modeling, that is after minimal data processing. The dimension reduction is performed on the 20 first principal components. Each point is a single cell colored by cell type and shaped according to the chromatographic batch. Resistant melanoma cells were characterized by Leduc et al. [18], but were modeled by scplainer as part of the main melanoma population to serve as positvie controls. Undefined melanoma cells are cells that were excluded by the authors but were included in this analysis. **b** Same as a. but the t-SNE was performed on the top 20 APCA+ results for the cell type effect. **c** Same as **b**, but colored by cluster as determined by *k*-means clustering ($k = 3$). **d** Biplot of the first two APCA+ components for the cell type effect. Points represent cells (PC scores) while arrows and labels highlight the top 40 peptides with the largest PC loadings

The first objective for using APCA+ is to assess the quality of the batch correction. As expected from the analysis of variance, a PCA on the unmodeled data reveals the presence of strong batch effects (based on 20 components that explain 62% total variance, Figs. 4a, S5a). The two cell types are separated based on the MS acquisition run and TMT label (Fig. S7). Conversely, APCA+ on the cell type effect shows that the modeling approach greatly removes the batch effects and perfectly separates the different cell types (based on 20 components that explain 29% total variance, Figs. 4b, S5b, S7). Despite the presence of weak residual batch effects (Fig. S7g), *K*-means clustering retrieves the expected cell populations (Figs. 4c, S8).

The second objective of APCA+ is to explore cellular heterogeneity and unknown source of variation. In agreement with the differential abundance analysis (Fig. 3b), APCA+ on the cell type indicates that monocytes are characterized by high intensities of CORO1A, LCP1, TMSB4X, and RPL13, while melanoma cells are characterized by higher intensities of VIM and MCALL1 (Fig. 4d). Moreover, we also find a small subpopulation of melanoma cells that segregate from the main population. Leduc et al. [18] have reported a subpopulation of melanoma cells primed for drug resistance after extensive data processing and experimental validation. We consider this subpopulation as an interesting positive control to assess whether scplainer can uncover unknown biological information. After *K*-means clustering, we find a strong overlap between the validated populations by Leduc et al. [18] and our clustering results (Fig. 4b, c). Refitting an scplainer model using this clustered cell partition, followed by protein set enrichment analysis, showed that the main melanoma population is enriched in proteins involved in metabolic processes, while the subpopulation is enriched in proteins involved in cellular respiration and oxidative processes (Figs. S9, S10), further supporting the conclusions from the original publication.

### Benchmarking batch correction against related approaches

One feature of scplainer is the removal of batch effects (Fig. 1b). Several approaches for batch correction have already been applied to SCP data. We will first compare the conceptual differences of each method with scplainer before benchmarking their performance on the data set from Leduc et al. [18].

To this date, ComBat [31] stands out as the most widely used approach for batch correction in SCP data [7]. While both scplainer and ComBat rely on linear modeling, there are notable distinctions between the two methods. First, ComBat applies a Bayesian framework to estimate the linear model, facilitating information sharing across peptides. This is particularly useful for experiments with limited sample size. Second, while scplainer aligns the means across batches, ComBat also includes a scale adjustment to account for differences in variance. Third, ComBat is designed to correct for a single source of batch effects, implying that it can only handle either MS acquisition effects or labeling effects. In the benchmarking described below, we will focus on the former as it accounts for the majority of the variance (Fig. 2a). Lastly, ComBat cannot deal with high proportions of missing values, hindering its application to SCP data without the use of imputation. Fortunately, HarmonizR [17] was specifically developed to handle missing values with ComBat through data dissection based on shared patterns of missing values. This enables batch correction with ComBat without relying on data imputation. We will refer to the batch correction approach using ComBat through HarmonizR as the HarmonizR-ComBat approach.

limma [15] is another approach that has been reported to perform batch correction in SCP data [18]. limma and scplainer are highly related approaches as they both rely on linear regression. The difference with limma is that scplainer deals with missing values by adapting the model for every peptide based on the pattern of missing values.

The batch correction performed by scplainer also resembles the batch correction applied by the RUV-III-C model [32]. Both approaches work in the presence of missing values and attempt to extract biological variation from technical variation. However, RUV-III-C estimates unknown latent variables that are considered an unwanted source of variation. This requires the presence of control peptides. A control peptide is a peptide that is not affected by biology and that is found across all cells. Unfortunately, the presence of control peptides poses a strong assumption that cannot be met for SCP experiments. We will therefore not include RUV-III-C in our benchmark.

Finally, scplainer attempts to meet similar needs to those covered by the scPROTEIN approach, a deep graph contrastive learning framework [33]. The two solutions are however fundamentally different. First, scPROTEIN uses a non-linear embedding that can accommodate complex relations and interactions between variables in the data, but is prone to overfitting. Complex deep-learning approaches may not be necessary for modeling single-cell data. Several benchmarking studies have demonstrated that simple methods often perform best in scRNA-Seq [34, 35]. Finally, the scPROTEIN framework does not allow for statistical inference and model exploration, leading to results that are difficult to interpret and validate. Unfortunately, we will not include scPROTEIN as we could not successfully integrate the author’s software with our hardware.

**Fig. 5:**
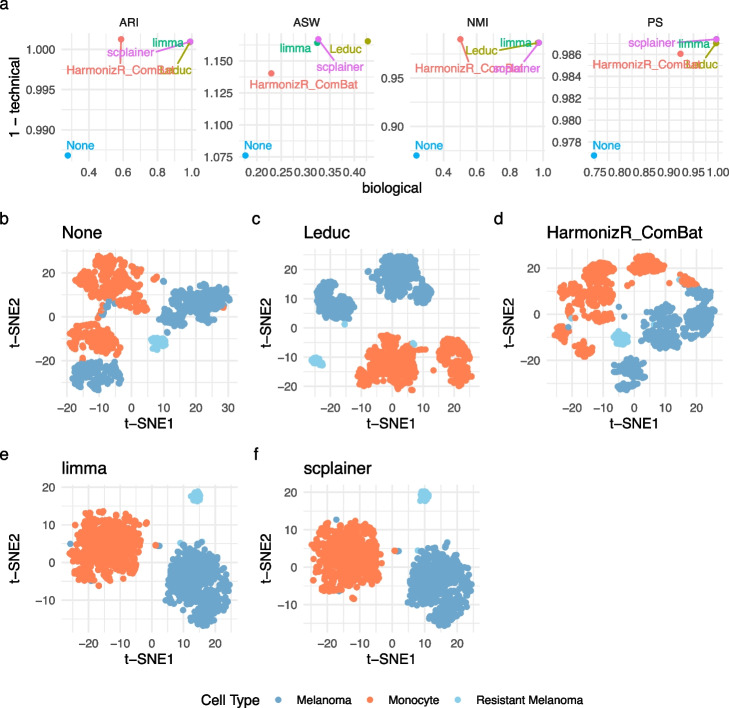
Benchmarking batch correction on the SCP data set by Leduc et al. [18] **a** Batch correction performance after the processing workflow by Leduc et al. [18] (Leduc), HarmonizR-Combat, limma and scplainer, as measured by 4 metrics (ARI, ASW, NMI, and PS). We include performance without batch correction (None) for baseline comparison. The biological performance is obtained by computing the metric while considering cell type labels. Higher values indicate a better separation of the biological groups. The 1-technical performance is obtained by computing the metric while considering the mixing of the MS acquisition runs. Higher values indicate a better mixing of the batch effects. **b**–**f** t-SNE results computed on the 20 first principal components. Each point is a cell and is colored based on the known cell type

We conducted a comprehensive benchmarking of scplainer against comparable batch correction approaches using the data set provided by Leduc et al. [18]. To assess the performance of batch correction, each method was applied to the peptide data generated after minimal processing. Subsequently, we performed PCA followed by *K*-means clustering. The PCA results and clustering results were used to compute the adjusted rand index (ARI), the normalized mutual information (NMI), the purity score (PS), and the average silhouette width (ASW) (Fig. 5a). Finally, we visually assessed cell type separation by performing t-SNE on the PC scores (Fig. 5b–f). We applied the same strategy to the data without batch correction to establish the baseline performance. We also included the data generated after the data processing carried out by Leduc et al. [18]. This complex workflow involves imputation, several iterations of normalization, two batch correction steps (reference alignment and limma), and peptide-to-protein aggregation.

The benchmarking results demonstrate an overall improvement in performance for all approaches compared to no batch correction (Fig. 5a). HarmonizR-ComBat showed a lower performance gain, while the remaining methods exhibited comparable performance. The visual assessment revealed that all methods were able to successfully segregate the expected cell types, although this was already noticeable for the uncorrected data (Fig. 5b–f). As anticipated, scplainer and limma resulted in similar dimension reduction results, as both approaches rely on linear regression. Upon closer inspection of the residual batch effects, each method exhibited different strengths and weaknesses (Fig. S11). The reduced performance gains by ComBat can be attributed to strong residual labeling effects that could not be explicitly accounted for. However, ComBat achieved excellent batch mixing within each label partition. The workflow by Leduc et al. [18] exhibit strong cell-type partitioning, but also a partitioning related to chromatographic batch. In contrast, limma and scplainer displayed a single main partition for each cell type, but with local differences between MS acquisition batches within each cell-type partition. Hence, scplainer demonstrated comparable performance to other batch correction approaches for SCP data, while it enhances model exploration and interpretable results. In other words, our decision to limit the data processing steps and create a flexible and principled approach that fits any SCP data set does not compromise the effectiveness of batch correction.

### Data integration

**Fig. 6:**
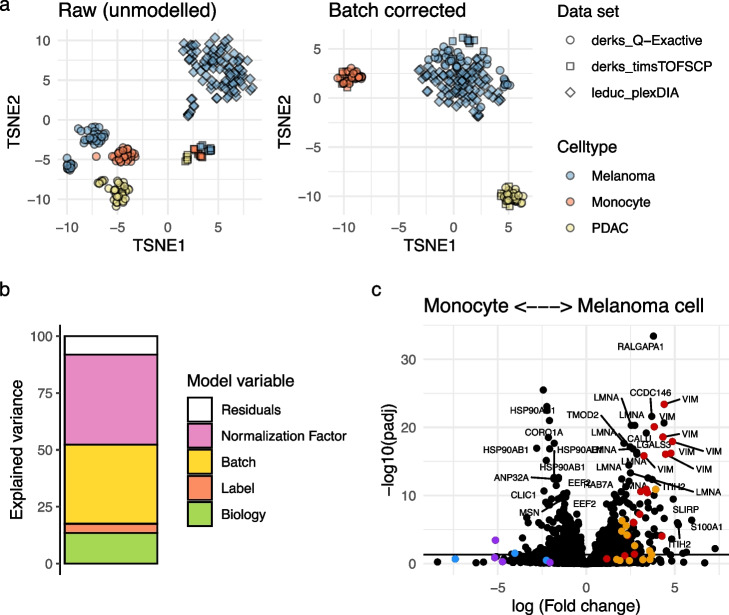
Integration of the plexDIA data sets by Leduc et al. [18] and Derks et al. [19] **a** t-SNE of the three data sets before integration. The dimension reduction is performed on the 20 first principal components for the data after minimal processing (left) or for the batch-correct data (right). Each point is a single cell and is colored based on the cell type and shaped according to the data set. **b** Analysis of variance for the integrated data sets. The normalization factor is the median intensity in a cell, the batch variable is the MS acquisition run, the label variable accounts for the mTRAQ-3 labeling effect and the biological variable models the cell type. **c** Volcano plot that shows the adjusted *p*-values against the log fold change between melanoma cells and monocytes. Each point represents a peptide. A positive log fold change indicates the abundance is higher in melanoma cells. We highlight the same peptides as in Fig. 3b: VIM (red), CTTN (orange), ARHGDIB (blue), and LCP1 (purple)

The ability of scplainer to perform batch correction makes it suitable for the integration of different data sets. To illustrate this, we apply scplainer to combine and explore three plexDIA data sets. The first data set contains only melanoma cells ($n = 106$) and was acquired by Leduc et al. [18] with a Q-Exactive instrument. The second data set contains melanoma cells ($n = 38$), monocytes ($n = 27$) and pancreatic ductal adenocarcinoma (PDAC) cells ($n = 33$) and was acquired by Derks et al. [19] with a Q-Exactive instrument. The last data set is similar to the second data set but was acquired on a timsTOF-SCP instrument and contains 9 melanoma cells, 10 monocytes, and 6 PDAC cells. As each of the three data sets comprises distinct cell types and was obtained by different experimenters, at various time points, and using different instruments, significant batch effects are present. However, despite these variations, the cell types are well separated in each data set. This observation indicates the presence of meaningful biological information to be extracted, as illustrated in Fig. 6. We build our model using the median cell intensity as a normalization factor, the MS acquisition run and the mTRAQ label as technical variables and the cell type as a biological variable. We can see the three cell types are separated in the resulting batch-corrected data and the three data sets aggregate within each cell type (Fig. 6a).

Similar to the analysis above (Fig. 2), the analysis of variance for the integrated data sets revealed that most of the variance is attributed to technical variables. However, 14% of the total variance is explained by the cell type (Fig. 6b), bearing meaningful biological information. When comparing monocytes to melanoma cells, proteins such as VIM, LMNA, CALU, LGALS3, CTTN, and CORO1A are strongly and confidently differentially expressed (Fig. 6c). To assess the consistency between the integration analysis and the analysis outlined above, we compared the differences between melanoma cells and monocytes obtained during both analyses. Out of the 2561 peptides that were modeled in both analyses, 874 peptides (34%) were consistently more abundant in monocytes and 803 (31%) were more abundant in melanoma cells. However, the two methods disagree for the remaining 884 peptides (35%). This is, for instance, the case of one of the LGALS1 peptides. Its abundance is higher in melanoma for the integration analysis but higher in monocytes for the first analysis. This pattern is not an artifact of the analysis, as it is already observed before data modeling (Fig. S12). Moreover, we noted variations in the amplitude of fold changes between the two analyses. For instance, peptides of the LCP1 and ARHGDIB proteins, which were highly differentially expressed in the first analysis (Fig. 3b), exhibited marginal significance in the integration analysis (Fig. 6d). These contradictory results may stem from fluctuations during cell culture, during cell preparation, during sample acquisition (DDA in the first analysis, DIA in the second), or data quantification. The standardized approach adopted in scplainer facilitated a straightforward and comprehensive comparison between the two analyses.

## Discussion

We presented scplainer, an approach that fulfills the current needs of SCP data analysis. First, we developed a minimal data processing workflow applicable to data generated by any SCP protocol, coupled with a flexible data modeling. We demonstrated the application of scplainer on a TMT-based DDA data set and an mTRAQ-based DIA data set. Furthermore, we provide a use case for a label-free data set on the SCP.replication website [7, 36]. Users can tailor their model specifications based on the available cell annotations. For example, labeling effects are incorporated for multiplexed data sets while excluded for label-free data sets.

Second, we demonstrated that scplainer effectively disentangles biological effects from technical effects, allowing for batch correction. Our results indicate that the batch correction carried out by scplainer is suitable for integrating data sets from different experiments. We further observed that the batch correction performance using scplainer is comparable to the best-performing methods currently used for SCP data. Additionally, scplainer is specifically designed to accommodate missing values thanks to adaptive modeling that accounts for the patterns of missing data. This also allowed the development of an objective filter for removing peptides with an excessive number of missing values relative to the number of model parameters to estimate.

Third, scplainer’s data modeling is based on linear regression, ensuring interpretable results. We introduce three distinct approaches that offer complementary insights into the data. The analysis of variance quantifies the contribution of each descriptor to the overall variance in the data. This analysis can also be conducted at the peptide level, hence helping the identification of peptides with pronounced biological effects. The differential abundance analysis assesses the statistical significance of differences in peptide intensities between two groups of interest. The component analysis delves into patterns of cellular heterogeneity, with a focus on specific biological or technical effects. These patterns are projected into a reduced dimension space, which can be used for visualization and downstream analysis.

Fourth, scplainer’s implementation is built on the SingleCellExperiment class [14], a widely used data container for single-cell data analysis. The data container serves as an interface to numerous downstream analysis algorithms dedicated to single-cell applications [14]. Therefore, the modeling results produced by scplainer can be readily investigated by these algorithms. Moreover, Warshanna and Orsburn [37] recently emphasized the importance of interactive tools for exploring SCP data. Data produced by scplainer can be seamlessly provided to iSEE, a powerful interactive tool crafted for in-depth exploration of single-cell data formatted using the SingleCellExperiment data structure [38].

Finally, scplainer is implemented in the R/Bioconductor scp package, an open-source package for manipulating and analyzing SCP data. By releasing our software through Bioconductor [39], we ensure a high standard of quality, as all submissions undergo a rigorous software review process. Additionally, Bioconductor mandates comprehensive documentation, and the software is subjected to weekly testing to ensure software integrity.

While we believe scplainer provides a comprehensive solution for SCP data analysis, we acknowledge several limitations that need further investigation. The first limitation is that scplainer implicitly assumes that missing values are missing completely at random (MCAR). A study by Li and Smyth [40] revealed that missing values in proteomics data are also missing not at random (MNAR), meaning the probability of detecting a peptide depends on its underlying intensity. However, we have previously found no evidence of a relationship between the proportion of missing values and the peptide intensity in SCP data [9]. Moreover, the authors demonstrated that the gain in accuracy from accounting for MNAR is minimal. Therefore, integrating an MNAR probability estimation in the model holds theoretical benefits, but, in practice, it may introduce an unnecessary computational burden with limited practical advantage.

Another limitation arises from the presence of residual batch effects after modeling with scplainer. After batch correction, cells within each population still cluster based on the TMT label and the acquisition run (Fig. S7g). Importantly, similar residual batch effects are also observed for other batch correction approaches (Fig. S11). Residual batch effects seen with scplainer may be attributed to the fact that TMT labeling effects may not be identical across different batches. One strategy to solve this issue is to include an interaction term between TMT labels and MS acquistion runs. However, such an approach would lead to a combinatorial increase in the number of parameters, resulting in an overspecified model.

scplainer’s modeling and statistical inference require that the sources of variation are independent. While a well-designed experiment should randomize different conditions across technical blocks [16], such designs are not always possible in practice, especially when considering biological replication. For example, in clinical studies, different treatment strategies are often tested on distinct sets of patients (i.e., the biological replicates), and each patient contains a distinct set of cells, leading to a hierarchical structure between the treatment descriptor and the patient descriptor. Using scplainer’s statistical inference framework without considering the expected increased correlation between cells within a patient would lead to inaccurate *p*-values. To analyze such hierarchical data, a linear mixed model could replace the linear regression model. Although linear mixed models have been developed for proteomics, such as the MSqRob model [41], their performance for single-cell applications still requires investigation.

## Conclusions

scplainer effectively uncovers meaningful biological information from complex SCP thanks to minimal data processing, principled modeling, and thorough exploration and visualization. scplainer corroborates previous findings that were obtained using complicated data processing workflows, showcasing that intricate data processing is unnecessary. Instead, our standardized approach enables future SCP experimenters to move their efforts from how to analyze the data to how to interpret the data.

## Methods

### Linear modeling

The linear model can be written as:

$$\begin{aligned} Y_{{O_j}j} = X_{{O_j}{S_j}} \beta _{{S_j}j} + \epsilon _{{O_j}j} \end{aligned}$$

$Y \in \mathbb {R}^{n \times m}$ is a matrix of intensities for *m* peptides in *n* single cells. $O_j$ denotes the set of cells for which the intensity is observed for peptide *j*, that is $O_j = \{i | Y_{ij} \in \mathbb {R}\}$. $X \in \mathbb {R}^{n \times p}$ is the model matrix. This matrix is constructed from the cell annotations and the model formula provided by the users. scplainer imposes an intercept, numerical variables are automatically centered, and categorical variables are encoded using sum contrasts. A categorical variable with *a* levels is therefore transformed to $a-1$ variables. To avoid confusion between the variables to model and the variables in the model matrix, we will refer to the former as model variables and the latter as model parameters. $S_j$ denotes the set of model parameters for peptide *j* that are not constant after subsetting the model matrix with $O_j$, that is ${S_j = \{k | var(X_{{O_j}k})> 0\}}$. The intercept is by definition a constant for all cells, so scplainer ensures the parameter index of the intercept is added to the set $S_j$. $\beta \in \mathbb {R}^{p \times m}$ is the matrix of coefficients to estimate. $\beta _{{S_j}j} \in \mathbb {R}^{{|S_j|} \times 1}$ is estimated for every peptide separately using ridge-penalized least square regression:

$$\begin{aligned} \arg \min _{\hat{\beta }_{{S_j}j}} || Y_{{O_j}j} - X_{{O_j}{S_j}} \beta _{{S_j}j} ||^2 + \lambda || \beta _{{S_j}j} ||^2 \end{aligned}$$

$\lambda = 10^{-3}$ is a small penalty constant to stabilize estimation when the *n*/*p* ratio for peptide *j* is close to 1. Finally, $\epsilon _{{O_j}j} \in \mathbb {R}^{n \times m}$ is the matrix of residuals containing the information that is not captured by the model. It is estimated as:

$$\begin{aligned} \hat{\epsilon }_{{O_j}j} = Y_{{O_j}j} - X_{{O_j}{S_j}} \hat{\beta }_{{S_j}j} \end{aligned}$$

$\hat{\epsilon }_{{O_j}j}$ is assumed to follow a normal distribution, that is $\hat{\epsilon }_{{O_j}j} \sim \mathcal {N}(0, \sigma _j^2)$ with $\sigma _j^2$ the residual variance for peptide *j*. Because each peptide *j* is modeled only with the cells in $O_j$ and the parameters in $S_j$, scplainer does not estimate all the elements of $\hat{\beta }$ and $\hat{\epsilon }$. In the following sections, we will assume that the data were modeled at the peptide level as suggested in the minimal processing workflow.

### Analysis of variance

Analysis of variance explores the decomposition of the variance across the different model parameters. Each model variable $f \in F$ is encoded by a set of model parameters $K_f$ for peptide *j*. We compute the regression sum of squares (SSR) in peptide *j* as follows:

$$\begin{aligned} SSR^{(f)} = || X_{{O_j}K_f} \hat{\beta }_{K_f j} ||^2 \end{aligned}$$

Similarly, we also compute the error sum of squares (SSE) for peptide *j*:

$$\begin{aligned} SSE = || Y_{{O_j}j} - X_{{O_j}{S_j}} \hat{\beta }_{S_j j} ||^2 = || \hat{\epsilon }_{{O_j}j} ||^2 \end{aligned}$$

Finally, we compute the percentage of variance explained by the variable *f* or by the residuals for peptide *j* as:

$$\begin{aligned} \%var^{(f)} = 100 \frac{SSR^{(f)}}{SSE + \sum _{l \in F} SSR^{(l)}} \end{aligned}$$

$$\begin{aligned} \%var^{residual} = 100 \frac{SSE}{SSE + \sum _{l \in F} SSR^{(l)}} \end{aligned}$$

Global analysis of variance, that is the analysis of variance for the whole data set, combines the results for all peptides by averaging the percentage of variance explained across peptides.

Similarly, we compute the percentage of variance explained for a protein *q* by averaging the percentage of variance explained for the set of corresponding peptides *P*. Formally,

$$\begin{aligned} \%var^{(f)}_q = \frac{1}{|P|} \sum _{j \in P} \%var_j^{(f)} \end{aligned}$$

$$\begin{aligned} \%var^{residual}_q = \frac{1}{|P|} \sum _{j \in P} \%var^{residual}_j \end{aligned}$$

### Differential abundance analysis

The key step for differential abundance analysis is estimating the uncertainty associated with the coefficients $\hat{\beta }$. This is performed by estimating the variance-covariance matrix. Since our approach makes use of an adaptive modeling strategy, we possibly estimate a different model for every peptide *j* and need to compute a separate variance-covariance matrix for each peptide. We set $X^{(j)} := X_{{O_j}{S_j}}$ to simplify notations. Following Cule et al. [42], the estimation of the variance-covariance matrix in linear ridge regression is:

$$\begin{aligned} \hat{\sigma }^{(j)2}_\beta = (X^{(j)T}X^{(j)} + \lambda \textbf{I})^{-1} X^{(j)T}X^{(j)} (X^{(j)T}X^{(j)} + \lambda \textbf{I})^{-1} \hat{\sigma }^2_j \end{aligned}$$

$\hat{\sigma }^2_j$ is the residual variance for peptide *j* and is estimated as $\hat{\sigma }^2_j = \frac{SSE}{\nu }$, where $\nu$ is the residual degrees of freedom.

We perform a *t*-test to determine the statistical significance between two groups of interest. Given a matrix of contrasts $L \in \mathbb {R}^{c \times p}$, were *c* is the number of contrasts to assess:

$$\begin{aligned} T = \frac{ L \beta _{:j}}{\sqrt{L \hat{\sigma }^{(j)2}_\beta L^T}} \end{aligned}$$

Under the null hypothesis, that is in the absence of differences between groups, we expect *T* to follow a t distribution with $\nu$ degrees of freedom, that is $T \sim t_{\nu }$. We compute the *p*-value associated with every value of *T* for all peptides and adjust for multiple testing for each contrast using the independent hypothesis weighting (IHW) [43]. Compared to the Benjamini-Hochberg FDR control, IHW enhances statistical power by assigning data-driven weights to each peptide. We assign the weights based on the estimated peptide intensity baseline with the rationale that peptides with a high baseline intensity are less noisy and hence should be less penalized during multiple testing.

We also provide functionality to aggregate the peptide level inference to protein level inference. First, we combine the peptide-level *p*-values using grouped hypothesis testing, as implemented by the metapod R package [44]. This package offers a variety of methods to group *p*-values using different hypothesis testing approaches: Fisher’s method, Simes’ method, Berger’s method, Pearson’s method, minimum Holm’s approach, Stouffer’s *Z*-score method, and Wilkinson’s method. For example, the null hypothesis of Fisher’s method is that all the null hypotheses at the peptide level are true. In other words, a protein will be considered significantly differentially abundant if at least one of its peptides is significantly differentially abundant. Conversely, Berger’s intersection union test will consider a protein as significantly differentially abundant if all its peptides are significant. Next, metapod also returns the representative peptides, that is the peptide that has been used to compute the protein *p*-value. scplainer defines the protein log fold change as the log fold change of the representative peptide.

### Component analysis

The component analysis used in our workflows follows the work by Thiel et al. [30] and is inspired by the limpca package [45]. The analyses are performed using APCA+ (extended ANOVA-simultaneous component analysis). For each model variable $f \in F$, APCA+ computes the effect matrix, denoted $\hat{M}^{(f)} \in \mathbb {R}^{m \times n}$. Residuals are then added to the effect matrix, leading to matrix $A^{(f)} \in \mathbb {R}^{n \times m}$. $A^{(f)}$ is next decomposed using PCA into a score matrix, *S*, and a loading matrix, *V*. Formally,

$$\begin{aligned} A^{(f)} = \hat{M}^{(f)} + \hat{\epsilon } = S V^T \end{aligned}$$

The loadings are constrained to $VV^T = \textbf{I}$ and the scores are constrained to $SS^T = \lambda \textbf{I}$ where $\lambda$ is the vector of eigenvalues. To deal with the missing values contained in $A^{(f)}$, scplainer performs PCA using the NIPALS algorithm.

Finally, we allow aggregating APCA+ results computed on peptide-level data to protein-level results. We perform aggregation on the loadings as these rotate the peptide space. We compute the loadings for protein *p* and variable *f* as the median loadings of the *P* peptides that constitute protein *p*. This approach breaks the mathematical assumptions of PCA, namely the protein loadings are no longer guaranteed to be orthogonal, and the variance is no longer guaranteed to be maximal in the first PCs. However, we believe this approach provides reliable dimension reduction results for exploring effects at the protein level. Furthermore, this approach is inspired by state-of-the-art scRNA-Seq workflows to pseudo-bulk analyses where single-cells are aggregated by subject [14, 46].

### Benchmarking

We benchmarked all batch correction methods with the following approach. First, we perform minimal data processing on the data set by Leduc et al. [18]. A performant batch correction approach should enhance the separation of biological groups while ensuring that technical groups are well-mixed. The biological descriptor used to assess biological separation is the cell type information provided by the authors: cells are either monocytes, melanoma cells or a subpopulation of resistant melanoma cells. The technical grouping consists of the MS acquisition runs ($n = 130$). For HarmonizR-ComBat, we removed the median intensity per cell before batch correction while the median intensity was included as a technical descriptor for limma and scplainer. Next, we performed PCA on the batch-corrected data and kept the 20 first PCs. Since the batch-corrected data contained missing values, we estimated the PCA using the NIPALS algorithm. PCA results were used to cluster cells using *K*-means clustering using a *k* of 3, that is the number of expected cell types. We then assessed the batch correction performance using two strategies: a metric-based evaluation and a visualization-based evaluation. For metric-based assessment, we include the adjusted rand index (ARI), the normalized mutual information (NMI) and the purity score (PS) that provide a measure of how accurately a clustering result matches expected labels. The expected labels are the cell types when assessing biological separation and the expected labels are the MS acquisition runs when assessing technical separation. We also include the average silhouette width (ASW) that evaluates the relatedness between cells from within and between partitions. We used the known biological and technical labels when assessing partitioning using ASW. For the visualization-based evaluation, we performed dimension reduction with t-SNE to project the 20 PCs into two dimensions.

### Code and data sets

The purpose of this work is to move efforts from dealing with technical aspects of SCP data analysis to focusing on answering biologically relevant questions. We, therefore, implemented our principled data analysis approach in the R/Bioconductor package scp [8, 47]. The package provides high-quality documentation with comprehensive demonstration tutorials, accessible from the package website: https://uclouvain-cbio.github.io/scp/articles/scp_data_modelling.html. We further provide example analyses for a variety of SCP data sets on the SCP.replication website [7, 36]. The software is designed to accommodate DDA and DIA data, labeled and label-free data. The scp package relies on the QFeatures class [48] that offers an interface to many data processing functions. The minimal processing workflow suggested in this work is fully supported by QFeatures. Furthermore, we have demonstrated that scp can reproduce the results of published SCP workflows [7, 8]. This means that our modeling approach can be used with any custom data processing workflow. Next to that, the scp package also relies on the SingleCellExperiment class [14], meaning that the results can be easily plugged into other methods for downstream analysis, such as cluster analysis or trajectory analysis.

All data were retrieved from the scpdata R/Bioconductor package [7, 49] (Table S1). The data package directly offers quantification values at the precursor, peptide and/or protein level.

#### leduc2022_pScoPE

The data set was published by Leduc et al. [18] in the third version of the preprint. We retrieve the quantified data at the peptide-to-spectrum match (PSM) level. The data set is acquired using TMT-18 multiplexing with prioritized DDA [50]. Any zero value in the data is considered missing. We next filter out any PSM that is matched to a decoy sequence or a contaminant protein, that has a sample-to-carrier ratio smaller than 5%, that has a spectral purity lower than 60%, and that has an associated identification FDR greater than 1%. Single cells are removed if they have less than 750 quantified peptides, their median log intensity falls outside (6, 8) or the median coefficient of variation (CV) within proteins is larger than 60% (we follow the CV definition suggested by Specht et al. [25]). We then aggregate the PSM data to peptide data based on the peptide sequence and summarize values for a peptide in a cell as the median of the peptide intensities. The data set includes 16,670 peptides and 1530 cells (773 WM989-A6-G3 melanoma cells and 757 U-937 monocytes). Finally, we log-transform the data before modeling. The variables used for the linear regression modeling are the median intensity in the cell (normalization factor), the TMT-18 label (technical variable), the MS acquisition run (technical variable), and the cell type (biological variable). Peptides with an *n*/*p* smaller than 3 were removed, hence keeping 6055 peptides to model. *K*-means clustering was performed using kmeans from the stats R package. We ran again the scplainer workflow, but using the K-means clusters instead of the cell type information. Uniprot protein identifiers were converted to gene names using the EnsDb.Hsapiens.v86 database [51] and we performed protein set enrichment analysis using the Gene Ontology biological process database retrieved from MSigDB [52].

#### leduc2022_plexDIA

The data set was published by Leduc et al. [18] in the fourth version of their preprint and their final publication. We retrieve the quantified data at the precursor level. The data set is acquired using mTRAQ-3 multiplexing with DIA [19]. Any zero value in the data is considered missing. We next filter out any PSM that is matched to a contaminant protein. FDR filtering is already performed by DIA-NN used by the authors to generate the precursor data. Single cells are removed if their median log intensity is smaller than 9.5. We then aggregate the precursor data to peptide data based on the peptide sequence and summarize values for a peptide in a cell as the median of the peptide intensities. The data set includes 2586 peptides and 106 cells (WM989-A6-G3 melanoma cells). Finally, we log-transform the data before modeling. The variables used for the linear regression modeling are the median intensity in the cell (normalization factor), the mTRAQ label (technical variable), and the MS acquisition run (technical variable). No biological variable is included. Peptides with an *n*/*p* smaller than 1 were removed, hence keeping 2010 peptides to model.

#### derks2022

The data set was published by Derks et al. [19] in the second version of their preprint and their final publication. We retrieve the quantified data at the precursor level. The data set is acquired using mTRAQ-3 multiplexing with DIA. The authors used either a Bruker timsTOF-SCP (25 cells) or a ThermoFisher Q-Exactive instrument (98 cells). Any zero value in the data is considered missing. We next filter out any PSM that is matched to a contaminant protein. FDR filtering is already performed by DIA-NN used by the authors to generate the precursor data. Single cells are removed depending on the instrument. For the Q-Exactive, cells are removed if they have less than 750 peptides and their median log intensity is smaller than 9.4. For the timsTOF-SCP, cells are removed if their median intensity is smaller than 6.5. We then aggregate the precursor data to peptide data based on the peptide sequence and summarize values for a peptide in a cell as the median of the peptide intensities. The data set includes 6913 peptides and 123 cells (47 WM989-A6-G3 melanoma cells, 37 U-937 monocytes, and 39 HPAF-II PDAC cells). Finally, we log-transform the data before modeling. The variables used for the linear regression modeling are the median intensity in the cell (normalization factor), the mTRAQ label (technical variable), the MS acquisition run (technical variable), and the cell type (biological variable). Peptides with an *n*/*p* smaller than 1 were removed, hence keeping 3614 peptides to model.

#### Integration analysis

The integration analysis used the leduc2022_plexDIA and the derks2022 data sets described above after minimal processing. The peptides were matched across data sets based on the peptide sequence and combined in a single data set. Once combined, the data include 5817 peptides and 228 cells. The variables used for the linear regression modeling are the median intensity in the cell (normalization factor), the mTRAQ label (technical variable), the MS acquisition run (technical variable), and the cell type (biological variable). Peptides with an *n*/*p* smaller than 1 were removed, hence keeping 4148 peptides to model.

## Data Availability

All peptide data were retrieved from the scpdata package available on Bioconductor. The data package directly offers quantification values at precursor, peptide and/or protein level. All data are released under a permissive Artistic 2.0 licence. The reference to each data set is provided in Table S1.

## Code availability

The scplainer methods described in this work is available as part of the scp package [47] released on Bioconductor. The code to reproduce the figures presented in the manuscript is available in a dedicated GitHub repository, which is archived on Zenodo [53]. A reproducible software environment is provided through a Docker image. The scp package, the data (accessed through the scpdata package), and the source code to reproduce the results are released under a permissive Artistic 2.0 licence.

## References

[1] Matzinger M, Mayer RL, Mechtler K. Label-free single cell proteomics utilizing ultrafast LC and MS instrumentation: a valuable complementary technique to multiplexing. Proteomics. 2023. 10.1002/pmic.202200162. 36806919 10.1002/pmic.202200162 PMC10909491

[2] Petrosius V, Schoof EM. Recent advances in the field of single-cell proteomics. Transl Oncol. 2022;27: 101556. 36270102 10.1016/j.tranon.2022.101556 PMC9587008

[3] Ctortecka C, Mechtler K. The rise of single-cell proteomics. Anal Sci Adv. 2021;2(3–4):84–94. 38716457 10.1002/ansa.202000152 PMC10989620

[4] Slavov N. Single-cell protein analysis by mass spectrometry. Curr Opin Chem Biol. 2021;60:1–9. 32599342 10.1016/j.cbpa.2020.04.018 PMC7767890

[5] Kelly RT. Single-cell proteomics: progress and prospects. Mol Cell Proteomics. 2020;19(11):1739–48. 32847821 10.1074/mcp.R120.002234 PMC7664119

[6] Truong T, Webber KGI, Madisyn Johnston S, Boekweg H, Lindgren CM, Liang Y, et al. Data-Dependent Acquisition with Precursor Coisolation Improves Proteome Coverage and Measurement Throughput for Label-Free Single-Cell Proteomics. Angew Chem Int Ed Engl. 2023:e202303415. 10.1002/anie.202303415 PMC10529037 37380610

[7] Vanderaa C, Gatto L. The current state of single-cell proteomics data analysis. Curr Protoc. 2023;3(1): e658. 36633424 10.1002/cpz1.658

[8] Vanderaa C, Gatto L. Replication of single-cell proteomics data reveals important computational challenges. Expert Rev Proteomics. 2021;18(10):835–43. 34602016 10.1080/14789450.2021.1988571

[9] Vanderaa C, Gatto L. Revisiting the thorny issue of missing values in single-cell proteomics. J Proteome Res. 2023;22(9):2775–84. 37530557 10.1021/acs.jproteome.3c00227

[10] Hu M, Zhang Y, Yuan Y, Ma W, Zheng Y, Gu Q, et al. Correlated Protein Modules Revealing Functional Coordination of Interacting Proteins Are Detected by Single-Cell Proteomics. J Phys Chem B. 2023;127(27):6006-14. 10.1021/acs.jpcb.3c00014. 10.1021/acs.jpcb.3c00014 37368753

[11] Schoof EM, Furtwängler B, Üresin N, Rapin N, Savickas S, Gentil C, et al. Quantitative single-cell proteomics as a tool to characterize cellular hierarchies. Nat Commun. 2021;12(1):3341. 34099695 10.1038/s41467-021-23667-y PMC8185083

[12] Zhu Y, Scheibinger M, Ellwanger DC, Krey JF, Choi D, Kelly RT, et al. Single-cell proteomics reveals changes in expression during hair-cell development. Elife. 2019;8: e50777. 31682227 10.7554/eLife.50777 PMC6855842

[13] Zappia L, Theis FJ. Over 1000 tools reveal trends in the single-cell RNA-seq analysis landscape. Genome Biol. 2021;22(1):301. 34715899 10.1186/s13059-021-02519-4 PMC8555270

[14] Amezquita RA, Lun ATL, Becht E, Carey VJ, Carpp LN, Geistlinger L, et al. Orchestrating single-cell analysis with bioconductor. Nat Methods. 2020;17(2):137–45. 31792435 10.1038/s41592-019-0654-x PMC7358058

[15] Ritchie ME, Phipson B, Wu D, Hu Y, Law CW, Shi W, et al. Limma powers differential expression analyses for RNA-sequencing and microarray studies. Nucleic Acids Res. 2015;43(7): e47. 25605792 10.1093/nar/gkv007 PMC4402510

[16] Gatto L, Aebersold R, Cox J, Demichev V, Derks J, Emmott E, et al. Initial recommendations for performing, benchmarking and reporting single-cell proteomics experiments. Nat Methods. 2023;20(3):375–86. 36864200 10.1038/s41592-023-01785-3 PMC10130941

[17] Voß H, Schlumbohm S, Barwikowski P, Wurlitzer M, Dottermusch M, Neumann P, et al. HarmonizR enables data harmonization across independent proteomic datasets with appropriate handling of missing values. Nat Commun. 2022;13(1):3523. 35725563 10.1038/s41467-022-31007-x PMC9209422

[18] Leduc A, Huffman RG, Cantlon J, Khan S, Slavov N. Exploring functional protein covariation across single cells using nPOP. Genome Biol. 2022;23(1):261. 36527135 10.1186/s13059-022-02817-5 PMC9756690

[19] Derks J, Leduc A, Wallmann G, Huffman RG, Willetts M, Khan S, et al. Increasing the throughput of sensitive proteomics by plexDIA. Nat Biotechnol. 2023;41(1):50–9. 35835881 10.1038/s41587-022-01389-w PMC9839897

[20] Petrosius V, Aragon-Fernandez P, Üresin N, Kovacs G, Phlairaharn T, Furtwängler B, et al. Exploration of cell state heterogeneity using single-cell proteomics through sensitivity-tailored data-independent acquisition. Nat Commun. 2023;14(1):5910. 37737208 10.1038/s41467-023-41602-1 PMC10517177

[21] Zhang Y. Pepdesc: a method for the detection of differentially expressed proteins for mass spectrometry-based single-cell proteomics using peptide-level information. Mol Cell Proteomics. 2023;22(7): 100583. 37236439 10.1016/j.mcpro.2023.100583 PMC10316082

[22] Plubell DL, Käll L, Webb-Robertson BJ, Bramer LM, Ives A, Kelleher NL, et al. Putting humpty dumpty back together again: what does protein quantification mean in bottom-up proteomics? J Proteome Res. 2022;21(4):891–8. 35220718 10.1021/acs.jproteome.1c00894 PMC8976764

[23] Grégoire S, Vanderaa C, Ruys SPD, Mazzucchelli G, Kune C, Vertommen D, et al. Standardised workflow for mass spectrometry-based single-cell proteomics data processing and analysis using the scp package. 2023. arXiv:2310.13598. 10.1007/978-1-0716-3934-4_14 38907155

[24] Kong W, Hui HWH, Peng H, Goh WWB. Dealing with missing values in proteomics data. Proteomics. 2022;22(23–24):e2200092. 36349819 10.1002/pmic.202200092

[25] Specht H, Emmott E, Petelski AA, Huffman RG, Perlman DH, Serra M, et al. Single-cell proteomic and transcriptomic analysis of macrophage heterogeneity using SCoPE2. Genome Biol. 2021;22(1): 50. 33504367 10.1186/s13059-021-02267-5 PMC7839219

[26] Woo J, Clair GC, Williams SM, Feng S, Tsai CF, Moore RJ, et al. Three-dimensional feature matching improves coverage for single-cell proteomics based on ion mobility filtering. Cell Syst. 2022;13(5):426-434.e4. 35298923 10.1016/j.cels.2022.02.003 PMC9119937

[27] Vasaikar SV, Savage AK, Gong Q, Swanson E, Talla A, Lord C, et al. A comprehensive platform for analyzing longitudinal multi-omics data. Nat Commun. 2023;14(1):1684. 36973282 10.1038/s41467-023-37432-w PMC10041512

[28] Demeulemeester N, Gébelin M, Caldi Gomes L, Lingor P, Carapito C, Martens L, et al. Msqrob2PTM: differential abundance and differential usage analysis of MS-based proteomics data at the posttranslational modification and peptidoform level. Mol Cell Proteomics. 2024;23(2): 100708. 38154689 10.1016/j.mcpro.2023.100708 PMC10875266

[29] Dermit M, Peters-Clarke TM, Shishkova E, Meyer JG. Peptide correlation analysis (PeCorA) reveals differential proteoform regulation. J Proteome Res. 2021;20(4):1972–80. 33325715 10.1021/acs.jproteome.0c00602 PMC8592057

[30] Thiel M, Féraud B, Govaerts B. ASCA+ and APCA+: extensions of ASCA and APCA in the analysis of unbalanced multifactorial designs. J Chemom. 2017;31(6): e2895.

[31] Johnson WE, Li C, Rabinovic A. Adjusting batch effects in microarray expression data using empirical Bayes methods. Biostatistics. 2007;8(1):118–27. 16632515 10.1093/biostatistics/kxj037

[32] Poulos RC, Hains PG, Shah R, Lucas N, Xavier D, Manda SS, et al. Strategies to enable large-scale proteomics for reproducible research. Nat Commun. 2020;11(1):3793. 32732981 10.1038/s41467-020-17641-3 PMC7393074

[33] Li W, Yang F, Wang F, Rong Y, Liu L, Wu B, et al. ScPROTEIN: a versatile deep graph contrastive learning framework for single-cell proteomics embedding. Nat Methods. 2024. 10.1038/s41592-024-02214-9. 38504113 10.1038/s41592-024-02214-9

[34] Ahlmann-Eltze C, Huber W. Comparison of transformations for single-cell RNA-seq data. Nat Methods. 2023;20(5):665–72. 37037999 10.1038/s41592-023-01814-1 PMC10172138

[35] Soneson C, Robinson MD. Bias, robustness and scalability in single-cell differential expression analysis. Nat Methods. 2018;15(4):255–61. 29481549 10.1038/nmeth.4612

[36] Vanderaa C, Gatto L. SCP.replication: Single-cell replication package. 2023. Website. https://uclouvain-cbio.github.io/SCP.replication. Accessed 05 Aug 2025.

[37] Warshanna A, Orsburn BC. SCP Viz – A universal graphical user interface for single protein analysis in single cell proteomics datasets. bioRxiv 2023.08.29.555397. 10.1101/2023.08.29.555397.

[38] Rue-Albrecht K, Marini F, Soneson C, Lun ATL. iSEE: interactive summarizedexperiment explorer. F1000Res. 2018;7: 741. 30002819 10.12688/f1000research.14966.1 PMC6013759

[39] Huber W, Carey VJ, Gentleman R, Anders S, Carlson M, Carvalho BS, et al. Orchestrating high-throughput genomic analysis with Bioconductor. Nat Methods. 2015;12(2):115–21. 25633503 10.1038/nmeth.3252 PMC4509590

[40] Li M, Smyth GK. Neither random nor censored: estimating intensity-dependent probabilities for missing values in label-free proteomics. Bioinformatics. 2023;39(5):btad200. 10.1093/bioinformatics/btad200. 10.1093/bioinformatics/btad200 PMC10174703 37067487

[41] Goeminne LJE, Sticker A, Martens L, Gevaert K, Clement L. MSqrob takes the missing hurdle: uniting intensity- and count-based proteomics. Anal Chem. 2020;92(9):6278–87. 32227882 10.1021/acs.analchem.9b04375

[42] Cule E, Vineis P, De Iorio M. Significance testing in ridge regression for genetic data. BMC Bioinformatics. 2011;12:372. 21929786 10.1186/1471-2105-12-372 PMC3228544

[43] Ignatiadis N, Klaus B, Zaugg JB, Huber W. Data-driven hypothesis weighting increases detection power in genome-scale multiple testing. Nat Methods. 2016;13(7):577–80. 27240256 10.1038/nmeth.3885 PMC4930141

[44] Lun A. Metapod: meta-analyses on *P*-values of differential analyses (version 1.12.0). 2023. Software package. 10.18129/B9.bioc.metapod.

[45] Thiel M, Benaiche N, Martin M, Franceschini S, Van Oirbeek R, Govaerts B. limpca: An R package for the linear modeling of high-dimensional designed data based on ASCA/APCA family of methods. J Chemometr. 2023;37(7):e3482. 10.1002/cem.3482.

[46] McCarthy DJ, Campbell KR, Lun ATL, Wills QF. Scater: pre-processing, quality control, normalization and visualization of single-cell RNA-seq data in R. Bioinformatics. 2017;33(8):1179–86. 28088763 10.1093/bioinformatics/btw777 PMC5408845

[47] Vanderaa C, Gatto L. scp: Mass Spectrometry-Based Single-Cell Proteomics Data Analysis (version 1.15.1). 2023. Software package. 10.18129/B9.bioc.scp.

[48] Gatto L, Vanderaa C. QFeatures: quantitative features for mass spectrometry data (version 1.11.1). 2023. Software package. 10.18129/B9.bioc.QFeatures.

[49] Vanderaa C, Gatto L. scpdata: Single-Cell Proteomics Data Package (version 1.13.0). 2024. Software package. 10.18129/B9.bioc.scpdata.

[50] Huffman RG, Leduc A, Wichmann C, et al. Prioritized mass spectrometry increases the depth, sensitivity and data completeness of single-cell proteomics. Nat Methods. 2023;20:714–22. 10.1038/s41592-023-01830-1. 10.1038/s41592-023-01830-1 PMC10172113 37012480

[51] Rainer J. EnsDb.Hsapiens.v86: Ensembl based annotation package (version 2.99.0). 2017. Software package. 10.18129/B9.bioc.EnsDb.Hsapiens.v86.

[52] Subramanian A, Tamayo P, Mootha VK, Mukherjee S, Ebert BL, Gillette MA, et al. Gene set enrichment analysis: a knowledge-based approach for interpreting genome-wide expression profiles. Proc Natl Acad Sci U S A. 2005;102(43):15545–50. 16199517 10.1073/pnas.0506580102 PMC1239896

[53] Vanderaa C, Gatto L. Zenodo archive of UCLouvain-CBIO/2024-scplainer (version 1.0). 2023. Code repository. 10.5281/zenodo.15788970.

